# Pancreatic Hyperenzymemia in Inflammatory Bowel Disease: Clinical Characterization and Outcomes from the European PANDORA Registry

**DOI:** 10.3390/pathophysiology33030044

**Published:** 2026-07-01

**Authors:** Salvatore Crucillà, Asia Berlato, Antonietta Gerarda Gravina, Anneline Cremer, Piotr Eder, Massimo Claudio Fantini, Stefano Festa, Daniela Pugliese, Andreas Blesl, Anna Viola, Chiara Viganò, Sophie Vieujean, Ioannis Koutroubakis, Edoardo Vincenzo Savarino, Lieven Pouillon, Mathieu Uzzan, Pierre Ellul, Marie Truyens, Federico Caldart, Rachele Ciccocioppo, Luca Frulloni, Maria Cristina Conti Bellocchi

**Affiliations:** 1 Gastroenterology and Endoscopy Unit, Department of Medicine, University of Verona, 37134 Verona, Italy; salvatore.crucilla@univr.it (S.C.); asiaberla@gmail.com (A.B.); federicocaldart94@gmail.com (F.C.);; 2 Division of Hepatogastroenterology, University of Campania “Luigi Vanvitelli”, Department of Precision Medicine, 81100 Naples, Italy; 3 Erasme Hospital, Department of Gastroenterology, Université Libre de Bruxelles, 1070 Brussels, Belgium; 4 Poznan University of Medical Sciences, Department of Gastroenterology, Dietetics and Internal Medicine, 60-355 Poznan, Poland; piotreder@ump.edu.pl; 5 University of Cagliari, Department of System Medicine—Gastroenterology, 09042 Monserrato, Italy; 6 San Filippo Neri Hospital, IBD UNIT, 00135 Rome, Italy; 7 Università Cattolica del Sacro Cuore Ospedale Gemelli Isola, Isola Tiberina Roma, CEMAD—IBD UNIT—Unità Operativa Complessa di Medicina Interna e Gastroenterologia, Dipartimento di Scienze Mediche e Chirurgiche, 00186 Rome, Italy; daniela.pugliese@policlinicogemelli.it; 8 Department of Internal Medicine, Division of Gastroenterology and Hepatology, Medical University of Graz, 8036 Graz, Austria; 9 University of Messina—IBD Unit, Department of Clinical and Experimental Medicine, 98125 Messina, Italy; annaviola88@gmail.com; 10 Fondazione IRCCS San Gerardo dei Tintori, Department of Gastroenterology, 20126 Monza, Italy; chiara.vigano@hotmail.it; 11 Gastroenterology Unit, CHU Liege Hospital, 4000 Liège, Belgium; 12 University Hospital Heraklion—Gastrenterology Clinic, 71110 Heraklion, Greece; ikoutroubakis@gmail.com; 13 Gastroenterology Unit, Department of Surgical, Oncological and Gastroenterological Sciences, University of Padua, 35128 Padua, Italy; edoardo.savarino@unipd.it; 14 Imelda GI Clinical Research Center, Imelda General Hospital, 2820 Bonheiden, Belgium; lieven.pouillon@imelda.be; 15 IBD Unit, Department of Gastroenterology, Mondor Hospital, APHP, 92110 Clichy, France; 16 Division of Gastroenterology, Mater dei Hospital, 2090 Msida, Malta; ellul.pierre@gmail.com; 17 Department of Gastroenterology and Hepatology, Ghent University Hospital, 9000 Ghent, Belgium; 18 University “G. d’Annunzio” of Chieti Pescara, Department of Medicine and Ageing, 66100 Chieti, Italy

**Keywords:** pancreatic hyperenzinemia, CAPH, inflammatory bowel disease, Crohn’s disease, ulcerative colitis

## Abstract

**Background and Aims:** Pancreatic hyperenzymemia in inflammatory bowel disease (IBD) is an under-recognized and challenging condition, as elevated pancreatic enzymes may arise from heterogeneous mechanisms and do not necessarily reflect true pancreatic disorder. This multicenter study aimed to characterize the clinical spectrum, diagnostic work-up, and outcomes of hyperenzymemia in IBD. **Methods**: This retrospective multicenter study was conducted within the PANDORA network, including 34 international IBD centers. For the present analysis, only centers providing patient-level data on pancreatic hyperenzymemia were considered. Collected variables included demographic, clinical, biochemical, imaging, and therapeutic data. Cases were categorized into three predefined phenotypes: chronic asymptomatic pancreatic hyperenzymemia (CAPH), reclassified acute pancreatitis not meeting Atlanta criteria (recAP), and autoimmune pancreatitis (AIP). **Results**: A total of 148 IBD patients with elevated pancreatic enzymes were included (CAPH 54.7%, RecAP 35.1%, AIP 10.1%). Ulcerative colitis (UC) accounted for 56.8% of cases and Crohn’s disease (CD) for 43.2%. Overall, 73.6% of patients were asymptomatic at the time of enzyme elevation. Marked hyperenzymemia (≥3× ULN) occurred in 22.3% of patients and was more frequent in RecAP than in CAPH or AIP (50.0%, 6.2%, and 13.3%, respectively). IBD was clinically active in 48.6% of patients, with higher rates in CD. Notably, the clinical meaning of hyperenzymemia differed across IBD phenotypes: in CD it was more often associated with active inflammation, whereas in UC it more frequently prompted advanced imaging and led to the identification of AIP. Imaging strategies differed significantly across phenotypes, and drug withdrawal was common in CAPH and RecAP but unnecessary in AIP. Only two rechallenges confirmed a drug-related mechanism. **Conclusions**: Pancreatic hyperenzymemia in IBD encompasses a spectrum of conditions with different implications. A phenotype-oriented diagnostic approach is essential to avoid misclassification and unnecessary treatment changes.

## 1. Introduction

Inflammatory bowel diseases (IBDs), comprising Crohn’s disease (CD) and ulcerative colitis (UC) have a high and increasing prevalence across Europe, with a well-recognized north–south gradient and rising incidence reported in several countries over recent decades [1,2]. These conditions are frequently associated with extraintestinal manifestations, among which pancreatic involvement is increasingly recognized. Pancreatic disorders reported in the IBD range from acute pancreatitis (AP), autoimmune pancreatitis (AIP), and chronic pancreatitis (CP) with exocrine pancreatic insufficiency (EPI), to subclinical biochemical abnormalities such as persistent asymptomatic elevation of pancreatic enzymes [3,4,5,6,7].

Pancreatic enzyme elevation in IBD may arise from heterogeneous mechanisms, spanning from benign idiopathic hyperenzymemia (CAPH) to structural pancreatic disease [8,9]. Drug exposure—particularly thiopurines, 5-aminosalicylic acid compounds, corticosteroids, and biologic agents—may also contribute, although distinguishing drug-related injury from coincidental hyperenzymemia remains challenging [10,11,12]. Importantly, asymptomatic hyperenzymemia may represent a benign biochemical phenomenon in a substantial proportion of patients, analogous to chronic asymptomatic pancreatic hyperenzymemia described in the general population [13,14,15,16].

A particular immune-mediated entity, type 2 AIP, is strongly associated with IBD and may present with isolated enzyme elevation, representing a key differential diagnosis [17,18]. However, available evidence is limited by small sample sizes, heterogeneous definitions, and the absence of standardized diagnostic pathways, leading to uncertainty regarding the need for imaging, treatment modification, or long-term monitoring [19,20,21]. Large, real-world cohorts are needed to distinguish benign biochemical abnormalities from conditions requiring targeted management and to inform evidence-based diagnostic strategies.

The European PANDORA (PANcreatic DisORders in inflammatory bowel diseAse) registry, a multicenter retrospective database across 34 IBD centers, provides a unique opportunity to address this knowledge gap [22]. Within this framework, we aimed to characterize the prevalence, clinical features, imaging findings, management strategies, and outcomes of pancreatic hyperenzymemia in IBD, including both idiopathic/asymptomatic cases and those associated with structural pancreatic disease.

## 2. Materials and Methods

### 2.1. Study Design

This study represents a sub-analysis of the PANDORA project, a retrospective multicenter European registry conducted over a 10-year period (January 2011–December 2020). The registry was structured in two sequential phases: an initial snapshot survey aimed at assessing local diagnostic and management practices and estimating the prevalence of pancreatic disorders in IBD, followed by the systematic collection of patient-level data through a dedicated REDCap electronic case report form. The study protocol was approved by the Ethics Committee of each participating center (coordinating center approval: Prog. N. 3767CESC, 20 April 2022) and endorsed by the Clinical Research Committee of the European Crohn’s and Colitis Organization (ECCO). Written informed consent was obtained in accordance with local regulations.

### 2.2. Study Population

All adult patients (≥18 years) with a diagnosis of IBD and documented pancreatic hyperenzymemia between January 2010 and December 2020 were included. Pancreatic hyperenzymemia was defined as serum amylase and/or lipase levels above the upper limit of normal on at least one occasion [23]. Patients were classified into three groups based on final expert adjudication: (i) Idiopathic/IBD-related CAPH; (ii) hyperenzymemia initially labeled as AP but not fulfilling revised Atlanta criteria [24] after review (“reclassified AP”, RecAP); and (iii) hyperenzymemia associated with AIP. Patients with confirmed AP were excluded from the primary analysis. The participating centers that contributed cases of pancreatic hyperenzymemia are listed in Supplementary Table S1.

### 2.3. Data Collection

For each patient, the following variables were collected: demographic data (age, sex, smoking and alcohol habits, family history); IBD-related data (disease type, Montreal classification, disease duration, extraintestinal manifestations, prior IBD-related surgery); CAPH-specific data (date of first detection, type of enzyme elevation, proposed pathogenesis, IBD activity at the time of CAPH, ongoing IBD therapy including drug name and dosage when available); imaging work-up (modality and findings); AIP-related data (fulfillment of International Consensus Diagnostic Criteria- ICDC) [25], steroid treatment, and response. management strategy (drug withdrawal or continuation); and clinical outcomes during follow-up (enzyme normalization, persistent elevation, or progression to AP). IBD activity at the time of CAPH was classified as active or in remission based on clinical, endoscopic, and/or biochemical parameters, according to the treating physician’s assessment.

All cases underwent centralized review by investigators with expertise in pancreatic disorders. Patients initially classified as acute pancreatitis were reassessed according to the revised Atlanta criteria, requiring at least two of the following: characteristic abdominal pain, pancreatic enzyme elevation greater than three times the upper limit of normal, and imaging findings consistent with acute pancreatitis. Cases not fulfilling these criteria were reclassified as RecAP. Similarly, AIP diagnoses were reviewed according to International Consensus Diagnostic Criteria (ICDC) whenever sufficient information was available.

### 2.4. Statistical Analysis

Results were expressed as mean ± standard deviation (SD) or median with inter-quartile range (IQR) for continuous variables, and as absolute frequencies and percentages for categorical variables. Comparisons between CD and UC patients were performed using Student’s *t*-test or Mann–Whitney U test for continuous variables, and the Chi-square test or Fisher’s exact test for categorical variables, as appropriate. Differences across the three predefined phenotypes (CAPH, RecAP, AIP) were assessed using one-way ANOVA test for continuous variables, with post hoc pairwise comparisons when indicated. The association between drug withdrawal and enzyme normalization, and between imaging findings and AP progression, was evaluated using Fisher’s exact test, with calculation of odds ratios (OR) and 95% confidence intervals (CIs). All tests were two-tailed, and a *p*-value < 0.05 was considered statistically significant. Statistical analyses were performed using Jamovi (version 2.6.24).

## 3. Results

The final hyperenzymemia cohort (n = 148) originated from 27 centers, including both centers that systematically entered CAPH cases (n = 18) and additional centers contributing patients reclassified from other pancreatic categories.

The total of 148 patients, included 81 CAPH (54.7%), 52 reclassified AP (35.1%), and 15 AIP-associated hyperenzymemia (10.1%). Crohn’s disease accounted for 48.6% of cases and ulcerative colitis for 51.4%. Baseline characteristics are reported in Table 1. Among the 18 centers that contributed CAPH cases, CAPH represented 0.23% of the IBD population under follow-up (81/35,655).

### 3.1. Clinical and Diagnostic Features at Hyperenzymemia Detection

IBD phenotype distribution differed across groups, with a trend toward a higher proportion of CD in recAP (61.5%) compared with CAPH (43.2%) and AIP (33.3%) (*p* = 0.054). Overall, 109 of 148 patients (73.6%) were asymptomatic at the time of enzyme elevation, including all CAPH cases (100%), 16 (30.7%) recAP patients, and 11 (73.3%) AIP patients.

Among symptomatic recAP individuals, 7 patients (4.7%) reported typical abdominal pain without fulfilling diagnostic criteria for AP, 7 (4.7%) had non-specific abdominal pain, 6 (4.1%) presented with nausea or vomiting, and 2 (1.4%) with diarrhea. In the AIP group, 2 patients (1.4%) reported unintentional weight loss, 1 reported jaundice, and 1 (0.7%) had dyspepsia.

Overall, 33 of 148 patients (22.3%) had enzyme elevations ≥3× ULN, with a different distribution across groups (CAPH 6.2%, reclassified AP 50.0%, AIP 13.3%). The remaining patients exhibited only mild elevations. Isolated lipase dosage accounted for 79.8% of cases, amylase elevation for 11.9%, and combined elevations for 8.3%. At the time of hyperenzymemia, IBD was clinically active in 48.6% of patients, with no significant difference across phenotypes (*p* = 0.180). Among these 44/76 (57.9%) CD and 30/72 (41.6%) (*p* = 0.048).

Regarding ongoing IBD therapy, 5-ASA was used in 41.2% of patients, thiopurines in 36.5%, corticosteroids in 14.9%, and biologics in 31.8%, while 10.1% were not receiving any IBD therapy. Ongoing treatment differed significantly among phenotypes (*p* = 0.0001), with AIP patients more frequently untreated compared with CAPH and recAP. Drug withdrawal was more frequent in recAP (78.8%), than CAPH (22.2%), and absent in AIP (*p* = 0.001). The distribution across groups and drug withdrawal strategy are reported in Table 2.

The likelihood of treatment discontinuation differed substantially among phenotypes, being more than three-fold higher in RecAP than CAPH (78.8% vs. 22.2%). Conversely, advanced imaging was universally performed in AIP patients but remained uncommon among CAPH patients.

#### 3.1.1. CAPH

Among CAPH patients, a presumed etiology was reported in a minority of cases: the cause was considered unknown in 54 patients (66.7%), attributed to drug exposure in 24 (29.6%), and to active IBD in 3 patients (3.7%). Imaging was not performed in 11 patients (13.6%); among those evaluated, 43 (53%) only underwent abdominal ultrasound, 15 (18.5%) CT, 16 (19.7%) MRI, and 1 (1.2%) EUS (Table 3).

Imaging findings were unremarkable in the vast majority of cases, with only 2 (2.4%) patients showing mild, non-specific pancreatic changes and 5 (6.1%) displaying anatomical variants. Despite the absence of symptoms or imaging abnormalities, IBD therapy was discontinued in 18 CAPH patients (22.2%). At the time of CAPH detection, 10 of these patients were receiving thiopurines: 8 were on azathioprine (mean dose 1.28 mg/kg/day; range 0.5–2.0 mg/kg/day) and 2 on 6-mercaptopurine (1.5 and 1 mg/kg/day). Active IBD was present in 6 of the 10 patients receiving thiopurines who discontinued therapy (4/8 on azathioprine and 2/2 on 6-mercaptopurine). The remaining eight patients were receiving mesalazine, which was discontinued. Following treatment discontinuation, hyperenzymemia normalized in 17 patients, while one patient on azathioprine showed no changes.

Overall, during a median follow-up of 36 months [IQR 12–72], 37 patients (45.7%) showed no change in enzyme levels (including one who had discontinued therapy), 43 (53.1%) achieved complete normalization, and 1 patient (1.2%) progressed to chronic pancreatitis, with a CFTR mutation subsequently documented on genetic testing.

#### 3.1.2. RecAP Patients

RecAP patients included individuals initially recorded as AP but not fulfilling the revised Atlanta criteria. Of these 52 RecAP cases, 16 (30.8%) were completely asymptomatic, 16 (30.8%) presented with non-specific symptoms (non-typical abdominal pain, nausea, vomiting or diarrhea), and 20 (38.4%) reported typical pancreatic pain but had enzyme levels <3× ULN with no imaging evidence of pancreatitis (either normal or not performed).

Among the 52 patients initially registered as AP but subsequently reclassified, imaging was not performed in 21 cases (40.4%). Among the remaining patients, 22 (42.3%) underwent abdominal ultrasound, 10 (19.2%) CT, and 6 (11.5%) MRI (Table 3). Imaging was normal in 21 patients (40.4%), whereas 9 (17.3%) showed peripancreatic edema and 1 (1.9%) had a small peripancreatic fluid collection.

In clinical practice, 41 of 52 patients (78.8%) had their suspected IBD-related medication withdrawn because of the initial suspicion of AP. The suspected drug was mesalazine in 2 patients (4.9%), azathioprine in 37 patients (90.2%), one antibiotic (2.4%), and escitalopram in one case (2.4%). Among the 37 patients receiving AZA, the mean dose was 1.56 mg/kg/day (range 0.5–2.5 mg/kg/day). AZA treatment duration was available for 31 patients, with a median duration of 1 month (IQR 0.69–1.00), a mean duration of 1.86 months, and a range of 0.23–12 months; most patients (22/31, 71%) had been exposed for ≤2 months, whereas five patients (16%) had markedly longer exposure durations. Two patients underwent drug rechallenge, both showing a reproducible increase in pancreatic enzymes. Most patients achieved complete clinical resolution, with recurrence documented in only two cases during a median follow-up of 84 months [IQR 34–132].

#### 3.1.3. AIP-Related Hyperenzymemia

Among the 15 patients with AIP-associated hyperenzymemia, ICDC were not applied in 5 cases, while 9 were classified as probable type 2 AIP and 1 as definite type 2 AIP. Serum IgG4 levels were normal in 14 patients and mildly elevated (<2 × ULN) in one. Imaging evaluation included CT in 6 (40%) patients, MRI in 14 (93.3%), and EUS in 5 (33.3%) (Table 3). Most cases showed diffuse pancreatic involvement (13/15), including two with a hypovascular rim, whereas focal disease was observed in two patients. Seven patients received corticosteroid therapy, achieving a 100% clinical and biochemical response. During follow-up (median 35 months, IQR 22–60), 13 patients showed no recurrence or disease progression, while two evolved toward chronic pancreatitis.

## 4. Discussion

Pancreatic hyperenzymemia in IBD patients is an under-recognized yet challenging clinical condition. Its interpretation is often complex, as elevated pancreatic enzymes may arise from heterogeneous mechanisms and do not necessarily reflect true pancreatic disorder. At present, the pathophysiological basis of pancreatic hyperenzymemia in IBD remains poorly defined, and available hypotheses are largely speculative [9]. Intestinal inflammation can increase mucosal permeability, facilitating systemic absorption of amylase and lipase without pancreatic injury. Systemic inflammation may also alter enzyme metabolism and clearance, leading to transient biochemical elevations [26]. In addition, non-pancreatic sources of amylase and lipase—including inflamed intestinal tissue, immune cells, and extra-pancreatic organs—may contribute to mild hyperenzymemia in IBD. This uncertainty reflects the current state of the field rather than a limitation of our dataset, as no validated molecular pathways or biomarkers exist to date. The retrospective design of PANDORA did not allow for advanced biomarker or mechanistic testing; however, the clinical granularity of this cohort provides a much-needed framework to guide real-world interpretation of enzyme abnormalities and to integrate a pancreatologist’s perspective into IBD care. In this large multicenter cohort of IBD patients with elevated pancreatic enzymes, we identified three distinct clinical phenotypes—the benign chronic hyperenzymemia (CAPH), the misclassified acute pancreatitis patients, which did not fulfil the Atlanta criteria due to enzyme value, atypical clinical presentation and/or absence of imaging findings (recAP), and AIP-related hyperenzymemia—each characterized by specific patterns of presentation, diagnostic work-up, and outcomes. Overall, our findings demonstrate that not all cases of hyperenzymemia in IBD are the same, and that biochemical abnormalities alone are insufficient to distinguish benign conditions from true pancreatic disease.

CAPH emerged as the most frequent and clinically benign phenotype. Its true prevalence in IBD, however, cannot be reliably estimated from this registry, as not all participating centers routinely measure pancreatic enzymes (only 18 of 34 centers contributed cases of isolated hyperenzymemia). All CAPH patients were asymptomatic, imaging was normal in nearly all cases, and enzyme elevations were generally mild. More than half achieved complete normalization during follow-up, and only one patient progressed to CP due to an underlying CFTR mutation [27]. Despite this reassuring profile, IBD therapy was discontinued in over 20% of CAPH patients, highlighting a tendency toward overtreatment driven by diagnostic uncertainty. From a clinical perspective, our findings challenge the common assumption that pancreatic enzyme elevation necessarily indicates pancreatic toxicity, thus triggering treatment modifications. Our data suggest that a proportion of CAPH cases may actually represent drug-related hyperenzymemia rather than a true benign biochemical phenotype, as supported by the normalization of pancreatic enzymes after treatment withdrawal in nearly all patients who discontinued therapy. However, Teich et al. [28] showed that mild hyperenzymemia does not appear to translate into worse outcomes, and discontinuing effective therapies based solely on biochemical abnormalities may be detrimental.

The RecAP group was also the most heterogeneous, likely reflecting a mixture of drug-related hyperenzymemia, CAPH-like presentations, and occasional AIP. Importantly, several cases were probably drug-related hyperenzymemia rather than acute pancreatitis, as suggested by the absence of imaging abnormalities and the reproducible enzyme increase observed in the two rechallenge cases. At the same time, many treatment discontinuations were labeled as drug-related despite exposure durations that were not compatible with a pharmacologic mechanism, indicating that some elevations were unlikely to be medication-induced. This combination of true drug-related cases, non-pancreatic hyperenzymemia, and misinterpreted biochemical abnormalities contributes to the apparent heterogeneity of the RecAP cohort. Rare cases of true AP may have been included solely due to expected human diagnostic error—either clinical misinterpretation or suboptimal imaging assessment—rather than representing a distinct biological phenotype. These findings underscore the need for strict adherence to diagnostic criteria to avoid unnecessary treatment changes. Educational initiatives and standardized diagnostic pathways may reduce this source of misclassification and improve consistency among centers.

AIP represented the smallest but most distinct subgroup. Among the 15 patients with AIP-associated hyperenzymemia, ICDC [25] were not applied in five cases, reflecting the complexity of these diagnostic standards in real-world practice. In type 2 AIP, a definite diagnosis requires histological confirmation, which is not always feasible, and most cases therefore remain classified as “probable.” Accurate application of ICDC would benefit from dedicated pancreatology consultation, particularly in centers with limited experience in AIP evaluation. In our study, patients frequently underwent advanced imaging, and MRI was used more often in this group than in any other, reflecting the intrinsic diagnostic complexity of AIP and the need for highly specific radiological assessment. This pattern suggests that a more systematic and advanced diagnostic work-up might have uncovered additional AIP cases, particularly among patients with unexplained hyperenzymemia or incomplete imaging evaluation. Unlike the other two phenotypes, most AIP patients were not receiving any ongoing IBD therapy at the time of enzyme elevation. This absence of treatment reduced the suspicion of drug-related pancreatitis and prompted clinicians to pursue a more extensive imaging-based diagnostic work-up, which probably facilitated the identification of AIP.

Beyond the three phenotypes, our data also suggest that the clinical meaning of pancreatic hyperenzymemia differs between CD and UC. In CD, enzyme elevation was more frequently associated with active intestinal inflammation and with higher enzyme values, supporting the hypothesis that increased intestinal permeability, systemic inflammatory activity, and drug exposure—particularly thiopurines—may contribute to transient biochemical abnormalities without underlying pancreatic injury [28]. This pattern aligns with the concept that hyperenzymemia in CD often represents an epiphenomenon of disease activity rather than true pancreatic involvement [29].

In UC, the scenario appears substantially different. Because inflammation is confined to the colon, mechanisms related to increased small-bowel permeability or transmural inflammation are less frequent. Instead, UC is the IBD phenotype most strongly associated with type 2 autoimmune pancreatitis, which may present with isolated enzyme elevation and minimal or dyspepsia or weight loss. In our cohort, this translated into a higher diagnostic yield of advanced imaging in UC patients and into the identification of AIP in individuals whose initial presentation was limited to hyperenzymemia. These findings suggest that, in UC, pancreatic enzyme elevation warrants greater diagnostic vigilance, and that cross-sectional imaging—preferably MRI—should be considered to exclude autoimmune pancreatitis or other structural pancreatic disorders.

Taken together, these findings highlight the need for a structured and phenotype-aware diagnostic approach to pancreatic enzyme elevation in IBD. Recognizing CAPH as a benign entity and avoiding overinterpretation of biochemical thresholds are essential steps to prevent unnecessary treatment changes. At the same time, our results indicate that a more systematic and appropriately targeted diagnostic work-up—including advanced imaging when clinically justified—may help identify unrecognized AIP or clarify ambiguous cases, ultimately improving the management of IBD patients with hyperenzymemia.

Future prospective studies should evaluate the natural history of CAPH using standardized imaging protocols, serial enzyme measurements, and biospecimen collection. Translational research investigating intestinal permeability, immune-mediated pancreatic involvement, and genetic susceptibility may help clarify the biological basis of pancreatic hyperenzymemia in IBD. Prospective mechanistic studies will be essential to complement these findings and to elucidate the pathways underlying pancreatic involvement in IBD. Finally, cost-effectiveness analyses are needed to define the most appropriate diagnostic strategy for asymptomatic patients. This study has several limitations. First, its retrospective design inherently limits the ability to control for diagnostic variability across centers. Moreover, the retrospective nature of the registry did not permit standardized biospecimen collection or molecular analyses that could have provided mechanistic insights. Second, the true prevalence of CAPH in IBD cannot be reliably estimated, as pancreatic enzymes are not routinely measured in all participating centers, and only 18 of 34 contributed cases of isolated hyperenzymemia. Third, 32 of the 148 patients (21.6%) did not undergo any pancreatic imaging, introducing heterogeneity and potentially concealing undiagnosed CAPH or unrecognized AIP. Furthermore, biochemical assays were not standardized across participating centers, and imaging strategies were largely physician-driven. These factors may have introduced additional variability in the characterization of pancreatic hyperenzymemia. Finally, some degree of misclassification, particularly within the RecAP cohort, may reflect the expected rate of human diagnostic error, related to clinical interpretation or imaging assessment, rather than true biological overlap among phenotypes.

## 5. Conclusions

In conclusion, pancreatic hyperenzymemia in IBD reflects substantial diagnostic heterogeneity, and enzyme-only abnormalities frequently lead to over-investigation and unnecessary treatment modifications. Our findings underscore the need for a more cautious and context-aware interpretation of pancreatic enzyme elevations to avoid misclassification and overtreatment.

## Figures and Tables

**Table 1:** Baseline characteristics of patients (N = 148) distributed across the three predefined phenotypes (CAPH, RecAP, AIP).

Variable	CAPH (N = 81)	RecAP (N = 52)	AIP (N = 15)	*p* Value
Sex Male Female	53 (65.4%) 28 (34.6%)	27 (51.9%) 25 (48.1%)	7 (46.7%) 8 (53.3%)	0.183
Mean age, years ± SD	49.2 ± 16.3	44.1 ± 15.6	41.9 ± 12.5	0.092
CD UC	35 (43.2%) 46 (56.8%)	32 (61.5%) 20 (38.5%)	5 (33.3%) 10 (66.7%)	0.054
Active IBD at enzyme elevation	45 (55.6%)	21 (40,4%)	6 (40%)	0.180

Data are expressed as n (%) or mean ± SD. CD: Crohn’s disease; UC: ulcerative colitis; SD: standard deviation; IBD: inflammatory bowel disease; CAPH: chronic asymptomatic pancreatic hyperenzymemia; RecAP: reclassified AP; AIP: autoimmune pancreatitis.

**Table 2:** Ongoing therapies, imaging work-up and drug withdrawal according to phenotype.

Variable	CAPH (N = 81)	RecAP (N = 52)	AIP (N = 15)	*p* Value
Ongoing treatment * No Yes *5-ASA* *AZA* *6-MP* Corticosteroids Biologics Anti-TNF Anti-IL12/23 Integrin receptor antagonist	7 (8.7%) 74 (91.3%) 37 (45.7%) 13 (16%) 3 (3.7%) 10 (12.3%) 32 (39.5%) 23 (71.9%) 4 (12.5%) 5 (15.6%)	0 (0%) 52 (100%) 21 (40.4%) 36 (69.2%) 2 (3.8%) 11 (21.2%) 11 (21.2%) 8 (72.7%) 2 (18.2%) 1 (9.1%)	8 (53.3%) 7 (46.7%) 3 (20%) 0 (0%) 0 (0%) 1 (6.7%) 4 (26.7%) 1 (25%) 0 (0) 3 (75%)	0.001
Drug withdrawal	18 (22.2%)	41 (78.8%)	0	0.001

* Patients could receive more than one concomitant therapy. CAPH: chronic asymptomatic pancreatic hyperenzymemia; RecAP: reclassified AP; AIP: autoimmune pancreatitis; AZA: azathioprine; 6-MP: 6-mercaptopurine; 5-ASA: 5-aminosalicylic acid.

**Table 3:** Distribution of pancreatic imaging modalities across the three clinical phenotypes.

Variable	CAPH (N = 81)	RecAP (N = 52)	AIP (N = 15)	*p* Value
No imaging	11 (13.6%)	21 (40.4%)	0 (0.4%)	*p* < 0.001
Ultrasound only	43 (53.1%)	17 (32.7%)	0 (0%)	
Advanced imaging (CT/MRI/EUS)	27 (33.3%)	14 (26.9%)	15 (100%)	

CT: computed tomography; MRI: magnetic resonance imaging; EUS: endoscopic ultrasound.

## Data Availability

The data supporting the findings of this study are available from the corresponding author upon reasonable request.

## Supplementary Materials

The following supporting information can be downloaded at https://www.mdpi.com/article/10.3390/pathophysiology33030044/s1. Supplementary Table S1. Participating centers and countries; Supplementary Table S2. Additional baseline characteristics of patients included (Montreal classification, risk factors, EIMs and disease duration). Abbreviations: AIP, autoimmune pancreatitis; CAPH, chronic asymptomatic pancreatic hyperenzymemia; CD, Crohn’s disease; EIMs, extraintestinal manifestations; IBD, inflammatory bowel disease; RecAP, reclassified acute pancreatitis; SD, standard deviation; UC, ulcerative colitis.

## Abbreviations

The following abbreviations are used in this manuscript:

CD	Crohn’s disease
UC	ulcerative colitis
IBD	inflammatory bowel disease
CAPH	chronic asymptomatic pancreatic hyperenzymemia
RecAP	reclassified AP
AIP	autoimmune pancreatitis
AZA	azathioprine
6-MP	6-mercaptopurine
5-ASA	5-aminosalicylic acid
CT	computed tomography
MRI	magnetic resonance imaging
MRCP	magnetic resonance cholangiopancreatography
EUS	endoscopic ultrasound
