# Supplementary material for: Pancreatic Hyperenzymemia in Inflammatory Bowel Disease: Clinical Characterization and Outcomes from the European PANDORA Registry
Source: Pathophysiology. 2026 Jul 1;33(3):44. doi: 10.3390/pathophysiology33030044 (PMC13397837; doi:10.3390/pathophysiology33030044)
Supplement: Supplementary file 1 [file pathophysiology-33-00044-s001.zip › pathophysiology-4386842-supplementary.pdf]

Supplementary files

Supplementary Table S1. Participating centers and countries

No.	Center denomination	Country
1	University of Verona – Department of Medicine, Gastroenterology and Endoscopy Unit	Italy
2	University of Campania "Luigi Vanvitelli", Division of Hepatogastroenterology	Italy
3	University of Cagliari, Department of System Medicine – Gastroenterology	Italy
4	San Filippo Neri Hospital, IBD Unit, Rome	Italy
5	Fondazione Policlinico Universitario Agostino Gemelli IRCCS / Gemelli Isola, CEMAD – IBD Unit, Rome	Italy
6	University of Messina – IBD Unit, Department of Clinical and Experimental Medicine	Italy
7	Fondazione IRCCS San Gerardo dei Tintori, Department of Gastroenterology, Monza	Italy
8	Gastroenterology Unit, Department of Clinical Medicine and Surgery, University Federico II of Naples, Naples	Italy
9	University of Padua, Gastroenterology Unit, Padua	Italy
10	Dipartimento Universitario Clinico di Scienze Mediche Chirurgiche e della Salute, Università degli Studi di Trieste, Italy	Italy
11	Azienda Provinciale per i Servizi Sanitari (APSS), Gastroenterology and Digestive Endoscopy Unit, Santa Chiara Hospital, Trento	Italy
12	CHU Liège Hospital, Gastroenterology, Liegi	Belgium
13	Erasme Hospital, Gastroenterology, Brussels	Belgium
14	Imelda GI Clinical Research Center, Imelda General Hospital, Bonheiden	Belgium
15	Ghent University Hospital, Department of Gastroenterology and Hepatology, Ghent	Belgium
16	Department of Internal Medicine, Division of Gastroenterology and Hepatology, Medical University of Graz, Graz, Austria	Austria
17	Mondor Hospital, APHP, Department of Gastroenterology, IBD Unit, Clichy	France
18	Amiens University Hospital, Université de Picardie, Amiens	France
19	General Hospital Heraklion, Gastroenterology Clinic	Greece
20	Department of Gastroenterology, University Hospital of Larisa, Larisa	Greece
21	Tzaneio General Hospital of Piraeus, Department of Gastroenterology, Piraeus	Greece
22	Mater Dei Hospital, Division of Gastroenterology	Malta
23	Poznan University of Medical Sciences, Department of Gastroenterology, Dietetics and Internal Medicine	Poland
24	Gastroenterology Department, Unidade Local de Saude do Alto Ave, Guimarães	Portugal
25	Gastroenterology Department. Hospital Universitario de La Princesa, Instituto de Investigación) Sanitaria Princesa (IIS-Princesa), Universidad Autónoma de Madrid (UAM), and Centro de Investigación Biomédica en Red de Enfermedades Hepáticas y Digestivas (CIBEREHD), Madrid	Spain
26	Pauls Stradins Clininic University Hopital Gastroenterology Center, University Hospital, Riga	Latvia

Supplementary Table S2. Additional baseline characteristics of patients included (Montreal classification, risk factors, EIMs and disease duration).

Variable	Overall (N=148)	CAPH (N=81)	RecAP (N=52)	AIP (N=15)
Male sex	87 (58.8%)	53 (65.4%)	27 (51.9%)	7 (46.7%)
Female sex	61 (41.2%)	28 (34.6%)	25 (48.1%)	8 (53.3%)
Age, years, mean ± SD	46.7 ± 16.0	49.2 ± 16.3	44.1 ± 15.6	41.9 ± 12.5
Disease duration, years, mean ± SD	13.6 ± 9.3	14.8 ± 9.8	13.4 ± 8.8	8.6 ± 6.8
Disease duration, years, median [IQR]	12 [7-20]	13 [7-21]	11 [8-16]	7 [2-14]
Crohn's disease	72 (48.6%)	35 (43.2%)	32 (61.5%)	5 (33.3%)
Ulcerative colitis	76 (51.4%)	46 (56.8%)	20 (38.5%)	10 (66.7%)
A1 (<16 years)	11 (7.4%)	5 (6.2%)	6 (11.5%)	0 (0.0%)
A2 (16-40 years)	94 (63.5%)	47 (58.0%)	35 (67.3%)	12 (80.0%)
A3 (>40 years)	43 (29.1%)	29 (35.8%)	11 (21.2%)	3 (20.0%)
L1 ileal (among CD)	22 (30.6%)	12 (34.3%)	9 (28.1%)	1 (20.0%)
L2 colonic (among CD)	13 (18.1%)	7 (20.0%)	5 (15.6%)	1 (20.0%)
L3 ileocolonic (among CD)	38 (52.8%)	17 (48.6%)	18 (56.2%)	3 (60.0%)
L4 upper GI (among CD)	5 (6.9%)	2 (5.7%)	2 (6.2%)	1 (20.0%)
B1 inflammatory (among CD)	41 (56.9%)	21 (60.0%)	18 (56.2%)	2 (40.0%)
B2 stricturing (among CD)	21 (29.2%)	11 (31.4%)	8 (25.0%)	2 (40.0%)
B3 penetrating (among CD)	11 (15.3%)	4 (11.4%)	6 (18.8%)	1 (20.0%)
Perianal disease (among CD)	7 (9.7%)	4 (11.4%)	3 (9.4%)	0 (0.0%)
E1 proctitis (among UC)	19 (25.0%)	10 (21.7%)	3 (15.0%)	6 (60.0%)
E2 left-sided colitis (among UC)	23 (30.3%)	14 (30.4%)	7 (35.0%)	2 (20.0%)
E3 extensive colitis (among UC)	34 (44.7%)	22 (47.8%)	10 (50.0%)	2 (20.0%)
Current smoker	25 (16.9%)	11 (13.6%)	13 (25.0%)	1 (6.7%)
Former smoker	21 (14.2%)	11 (13.6%)	7 (13.5%)	3 (20.0%)
Never smoker	102 (68.9%)	59 (72.8%)	32 (61.5%)	11 (73.3%)
No alcohol consumption	118 (79.7%)	65 (80.2%)	43 (82.7%)	10 (66.7%)
Former drinker	3 (2.0%)	0 (0.0%)	2 (3.8%)	1 (6.7%)
Mild drinker	27 (18.2%)	16 (19.8%)	7 (13.5%)	4 (26.7%)
Heavy drinker	0 (0.0%)	0 (0.0%)	0 (0.0%)	0 (0.0%)
No family history	127 (85.8%)	74 (91.4%)	41 (78.8%)	12 (80.0%)
Family history of IBD	19 (12.8%)	6 (7.4%)	11 (21.2%)	2 (13.3%)
Family history of pancreatic disease	2 (1.4%)	1 (1.2%)	0 (0.0%)	1 (6.7%)

Family history of both IBD and pancreatic disease	0 (0.0%)	0 (0.0%)	0 (0.0%)	0 (0.0%)
Any EIM	34 (23.0%)	16 (19.8%)	14 (26.9%)	4 (26.7%)
No EIMs	114 (77.0%)	65 (80.2%)	38 (73.1%)	11 (73.3%)
Arthropathy	19 (12.8%)	9 (11.1%)	8 (15.4%)	2 (13.3%)
Metabolic bone disease	2 (1.4%)	2 (2.5%)	0 (0.0%)	0 (0.0%)
Eye disease	1 (0.7%)	0 (0.0%)	0 (0.0%)	1 (6.7%)
Oral disease	2 (1.4%)	1 (1.2%)	0 (0.0%)	1 (6.7%)
Primary sclerosing cholangitis	5 (3.4%)	2 (2.5%)	3 (5.8%)	0 (0.0%)
Skin disease	8 (5.4%)	3 (3.7%)	3 (5.8%)	2 (13.3%)
Urogenital disease	3 (2.0%)	2 (2.5%)	0 (0.0%)	1 (6.7%)
Other EIMs	1 (0.7%)	0 (0.0%)	1 (1.9%)	0 (0.0%)

Abbreviations: AIP, autoimmune pancreatitis; CAPH, chronic asymptomatic pancreatic hyperenzymemia; CD, Crohn's disease; EIMs, extraintestinal manifestations; IBD, inflammatory bowel disease; RecAP, reclassified acute pancreatitis; SD, standard deviation; UC, ulcerative colitis.
